# Barriers Broken: Genetic swamping in restored Brook Trout populations

**DOI:** 10.1371/journal.pone.0327926

**Published:** 2025-12-26

**Authors:** Rebecca J. Smith, Matt A. Kulp, Benjamin M. Fitzpatrick

**Affiliations:** 1 Department of Ecology and Evolutionary Biology, University of Tennessee, Knoxville, Tennessee, United States of America; 2 Great Smoky Mountains National Park, 107 Park Headquarters Road, Gatlinburg, Tennessee, United States of America; University of Tehran, IRAN, ISLAMIC REPUBLIC OF

## Abstract

Scientists use reintroductions to restore native species to their historical ranges but sometimes overlook effects of dispersal on genetic structure of restored populations. Unidirectional or biased gene flow can result in genetic swamping, where unique variation in a recipient population is replaced by genotypes from the source population. In theory, this can result in loss of advantageous alleles and adaptive capacity. In Great Smoky Mountains National Park native Brook Trout (*Salvelinus fontinalis)* are being restored to streams from which they had been extirpated. Multiple source lineages are mixed in restoration sites to maximize genetic diversity. However, in our study system, translocated fish were released unevenly along a rugged mountain stream, resulting in an upstream population coming from only one source stock and a downstream population that was a mixture of three source stocks. A natural cascade allows downstream dispersal but prevents or constrains upstream movement. Theory predicts that such biased movement will lead to genetic swamping, i.e., reduction or loss of representation of ancestral lineages released only in the downstream population. However, the rate of gene flow and degree of asymmetry were unknown. Here, we used genetic and population density data to confirm the directionality of dispersal, estimate the rate of genetic swamping, and assess alternative mitigation strategies. Our results indicate that the downstream population has already become dominated by ancestry from the upstream source and translocation from within the restored stream will not achieve the intended genetic diversity. Instead, introducing additional fish from the original source stocks above the natural barrier would be necessary to equalize the contribution of all three source populations. Our results emphasize the importance of understanding the interplay between dispersal and genetic structure for conservation planning.

## Introduction

Genetic diversity is crucial for a population’s resilience, as it increases the chance that some individuals will have traits that help them survive environmental changes [1–3]. However, growing threats like climate change, environmental disasters, and habitat fragmentation are disrupting the genetic variation of wildlife and fish populations. This puts species at risk by hindering their ability to adapt [4,5]. To combat these challenges, genetic management strategies focus on intentionally maintaining or boosting genetic diversity [4].

Species reintroductions are a powerful tool for boosting genetic diversity and promoting population resilience. These efforts work to restore populations to areas where they have been locally extirpated [6,7]. Since threatened species often exist in small, isolated populations, managers frequently use multiple sources for a single reintroduction site to prevent the depletion of any one source population [3,8]. This mixing of source populations is also intended to enhance genetic diversity, giving the restored population a greater potential to adapt to novel conditions [9].

In the absence of specific information on the distribution of advantageous traits in source populations, aiming for equal genetic contributions from each source is a measurable goal that will tend to maximize overall genetic diversity, and thus presumably adaptive potential [4]. Maximization of diversity, however, can be impacted by several factors [4,10]. For example, if one source has a distinct advantage in the early generations of admixture, its ancestry may come to predominate, which would not be detrimental [11]. Conversely, biases created inadvertently by human intervention or idiosyncrasies of the landscape might promote genetic swamping – defined as an undesirable over-representation of a genotype or ancestral lineage in an admixed population [12]. This process undermines the management goal of maximizing genetic variance and the efficiency of natural selection [13]. Genetic swamping can result from an intrinsic fitness advantage, unbalanced initial admixture proportions, or biased dispersal [14–17].

Brook Trout (*Salvelinus fontinalis*) are native to eastern North America, from the Southern Appalachian Mountains to southwestern Canada [18]. They have experienced significant population declines and extirpations due to habitat loss and competition with nonnative salmonids [18]. The extensive decline of Brook Trout across their native range has prompted numerous reintroduction efforts. In the Great Smoky Mountains National Park (GRSM), a Brook Trout reintroduction program aims to restore the species to their historical range, where they have been locally extirpated [19,20]. A barrier, whether natural or artificial, is an essential component of these reintroduction efforts to prevent the recolonization of nonnative trout. However, such barriers also prevent upstream dispersal of Brook Trout and might inhibit the connectivity and adaptability of reintroduced populations. This assumed one-way nature of downstream dispersal, combined with potentially uneven stocking, raises concerns about potential genetic swamping, which threatens to reduce the overall genetic diversity of the restored population and might undermine the project’s long-term success.

Here, we investigate the dynamics of genetic swamping in a recently restored Brook Trout population where a natural waterfall prevents upstream but not downstream dispersal. We use basic population genetics theory and a species-specific simulation model incorporating demographic data to estimate the dispersal rate and evaluate the likely effects of alternative approaches to mitigate genetic swamping.

## Methods

### Study System

**Fig 1:**
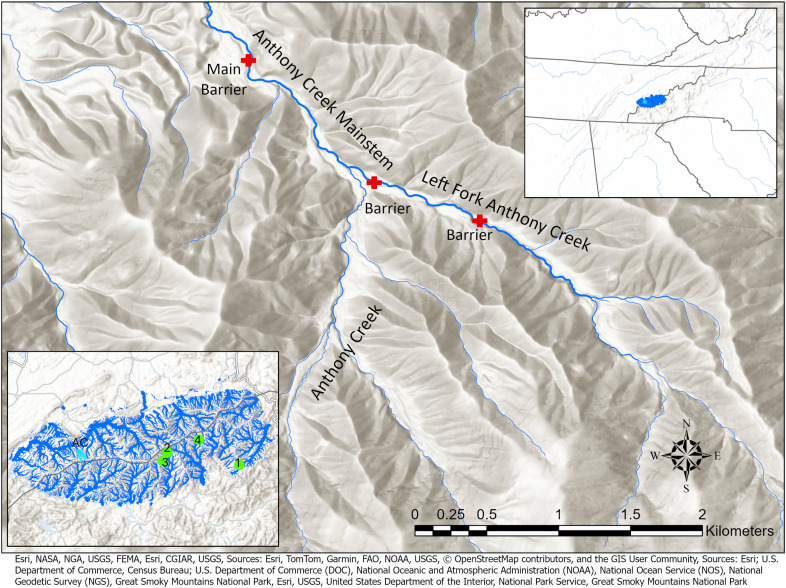
A map of Anthony Creek restoration site within The Great Smoky Mountains National Park. The main panel displays the Anthony Creek watershed within the Great Smoky Mountains National Park (GRSM). Red crosses mark the locations of in-stream barriers, and stream line width is proportional to stream order (mainstem and Left Fork Anthony Creek are both third-order streams). The top-right inset highlights Anthony Creek (cyan) within GRSM (blue), located across eastern Tennessee and western North Carolina in the Southeastern United States. The bottom-left inset shows the broader stream network of GRSM, with the Anthony Creek restoration site highlighted in cyan (labeled ‘AC’). Source streams are marked with green circles and numbered: 1. Bunches Creek, 2. Deep Creek, 3. Sahlee Creek, 4. Bearwallow Creek. All depicted source streams and Anthony Creek are located within the Little Tennessee watershed. Map contains information from OpenStreetMap and OpenStreetMap Foundation, which is made available under the Open Database License.

**Fig 2:**
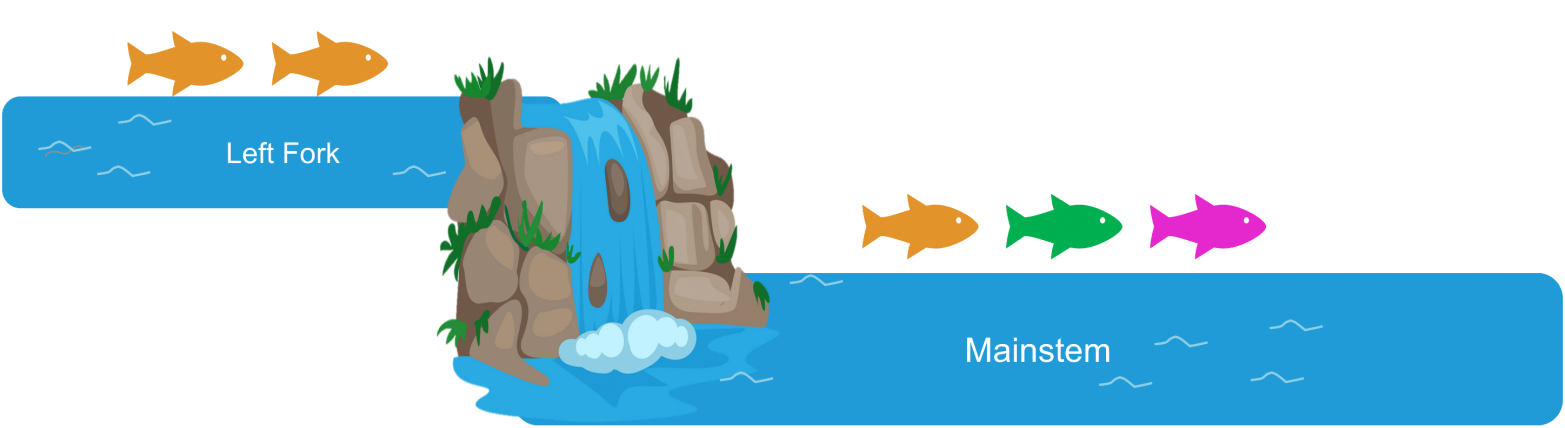
A schematic illustration of the translocation and stocking scenario of Anthony Creek. Fish from Bunches Creek (orange) were stocked in the Left Fork of Anthony Creek. Fish from Bunches (orange), Deep Creek (green), and Sahlee Creek (pink) were stocked in the mainstem, with a barrier preventing upstream dispersal between the two segments. This scenario illustrates how the Left Fork is expected to maintain primarily Bunches ancestry, whereas the mainstem was seeded with a mix of three sources, creating different ancestry dynamics across the system. Part of figure was created with Biorender.com.

This study focuses on a key case in GRSM at Anthony Creek (Fig 1), where a reintroduction used genetically diverse fish from three source populations. The creek’s unique physiography, which includes both natural and human-made barriers, influenced the reintroduction strategy. The unintentionally uneven distribution of fish from different source populations and the assumed one-way nature of downstream dispersal raised concerns about potential genetic swamping (Fig 2).

Anthony Creek (Figs 1-2) did not have extant populations of Brook Trout before reintroductions, and nonnative trout were removed before translocation [19]. Based on earlier genetic investigation [20] sources for translocation were chosen from accessible, high-density populations within the same major watershed (Little Tennessee River, Fig 1) rather than straight line distance from the target site. The management goal is to balance the preservation of source population numbers while achieving a target translocation density of 125–150 Brook Trout per kilometer at the restoration site [19,21,22].

Anthony Creek was initially stocked with 269 individuals from Bunches Creek, with the translocated fish distributed between the left fork (n = 135) and the mainstem (n = 134) of Anthony Creek. The fish were intended to be placed solely within the mainstem but were instead distributed between both areas due to miscommunication between field crews. In 2018, fish were translocated from Deep Creek (n = 135) and Sahlee Creek (n = 102) into the mainstem of Anthony Creek (Table 1). Management was unaware that fish had been stocked in the left fork until 2022. During that summer, we collected DNA samples from the three source populations, as well as from both the left fork and mainstem of Anthony Creek.

**Table 1:** Overview of populations included in the study, with the corresponding number of samples (N) collected from each population and the year of sampling.

Population	Sampled Year	N
Anthony Creek (left fork)	2022	29
Anthony Creek (mainstem)	2022	64
Bunches Creek	2022	100
Deep Creek	2022	98
Sahlee Creek	2022	60

Since the time of sampling, the composition of the Anthony Creek restoration has been made more complicated by an additional translocation of Brook Trout to a third stream segment. This segment is above the left fork segment and separated from it by another natural cascade. The segment was stocked with even mixture of fish from Bunches Creek and Bearwallow Branch. Thus, consideration of future actions will have to account for this most upstream population. Here we use our data from the left fork and main stem to test the hypothesis of unidirectional dispersal and estimate the rate of downstream immigration to understand the consequences of the spatially structured translocation history, and the feasibility of hypothetical interventions to maximize long-term genetic diversity.

### Sample Data & Processing

We captured live fish during regularly scheduled abundance surveys and removed adipose fin clips using sterile scissors prior to re-releasing fish alive within 100 meters of capture [21,23]. All procedures complied with the Animal Welfare Act and American Fisheries Society Guidelines for the use of fish in research [24]. Fin clip samples were collected by National Park Service staff for management and conservation purposes under approval of Scientific Research and Collecting Permit GRSM-SCI-2158. Our methods conform to ARRIVE guidelines. We extracted DNA from tissues using the Qiagen DNAeasy blood & tissue kits.

The Idaho Department of Fish and Game Eagle Genetics Lab performed the sequencing using a targeted sequencing panel of 304 loci developed by the lab in collaboration with the Columbia River Inter-Tribal Fish Commission for Brook Trout. The panel includes loci from Sauvage et al. 2012 [25] and additional loci identified through restriction site-associated DNA sequencing (Columbia River Inter-Tribal Fish Commission, unpublished data; IDFG, unpublished data). The panel was developed from Brook Trout populations in the northern portion of their range, far from our study area. Thus, there is little risk of ascertainment bias with respect to variability in our populations [26]. Further, ascertainment bias should have little to no impact on our research question, which uses reference samples from known source populations as the basis for evaluating their contributions to mixed populations [27,28]. The loci in the panel were not selected for a specific function. The dataset includes SNPs from both putatively neutral and functional regions of the genome [25]. Sample preparation and sequencing followed Genotyping in Thousands Sequencing (GTseq) protocols [29]. The Idaho Department of Fish and Game originally designed the targeted sequencing panel to score predefined SNPs in the Columbia River. However, we used the complete sequence data to discover and score all SNPs in our GRSM samples. IDFG filtered out low-quality reads and adapter contamination. We mapped the filtered and demultiplexed 79 base pair reads to the reference sequences using BWA mem [30]. We called variants using bcftools mpileup in the samtools suite [31], excluding those with a quality score below 200 or a depth below 20. Next, we examined the resulting VCF file manually in R [32] and tailored a quality control protocol to include additional filtering steps specific to our dataset as follows.

Initial filtering steps included the removal of three individuals identified as Rainbow Trout (*Oncorhynchus mykiss)*. Indels and invariant SNPs were excluded from the dataset using the extract.indels and is.polymorphic functions from the vcfR package [33], retaining only polymorphic SNP loci. We removed individuals with >5% missing genotypes. After these filtering steps, fewer than 20% of SNPs had still any missing data in any individual. To ensure a final dataset with no missing data we excluded all of them to produce a data set with no missing data across all individuals. A complete data matrix is required for multivariate analyses such as PCA and DAPC [34], which are important parts of our workflow. Collectively, the foregoing steps eliminated approximately half of the original 304 loci. The filtered VCF was converted to a genind object for multivariate analysis. We visually inspected the data with PCA plots which revealed clustering patterns associated with sequencing plates, suggesting potential batch effects. To mitigate this, we implemented a DAPC-based feature selection approach using the snpzip function in the glmnet package [35] to identify loci strongly associated with plate identity. A total of 48 SNPs were flagged and removed, effectively eliminating batch structure in the data. We then selected the first SNP from each locus to preserve the assumption of independence for ancestry estimation. The final filtered dataset included high-confidence genotype data for 232 alleles across 116 SNP loci. All sequencing data are available online under NCBI BioProject ID PRJNA127654. Additional materials are provided in S1 Dataset.

### Ancestry and migration rate estimation

To determine the relative contribution of each source to the restored populations in Anthony Creek, we estimated ancestry proportions for each sample using HIest [36]. We estimated source population allele frequencies from the source population samples and used these to estimate the ancestry proportions in Anthony Creek left fork and mainstem (Additional source and restored population genetic diversity statistics can be found in S1 Table). The threeway function estimates the most likely set of three ancestry proportions for each individual given its genotype and the given source allele frequencies. We sampled only 5 years after mixing, roughly two generations (the fourth breeding season). After only four breeding seasons, we expect a mixture of predominantly parental, F1, F2, and backcross genotypes because few if any F2 and backcrosses would have reached sexual maturity by the time of sampling [37]. If the first F1s were spawned as soon as possible in 2018, some could have been sexually mature by 2020, and their offspring (F2 and backcrosses) could be sexually mature by the 2022 breeding season, but that would be after our sample collection. It is extremely unlikely that we would have sampled F3s or later generation backcrosses. If there was no dispersal between restored stream segments, we would expect all samples from the left fork (above the cascade) to have 100% Bunches Creek ancestry, and samples from the mainstem to be a mixture with approximately 42% Bunches Creek ancestry, resembling the input proportions from each source. If there has been only downstream dispersal and no differences in fitness, the expected proportion of Bunches Creek ancestry in the mainstem would be a function of the immigration rate and time [10]:


$$A_{t+1}=A_{t}\left(1-m\right)+1.0\times m$$ (eq 1)

where *A*_*t*_ is the Bunches ancestry proportion in the mainstem at time *t*, *m* is the immigration rate per time interval, and 1.0 is the ancestry proportion of immigrants. This equation models expected change in ancestry proportions from year to year, assuming a constant immigration fraction *m*. The general solution


$$A_{t}=1-(1-A_{0}){\left(1-m\right)}^{t}$$ (eq 2)

predicts the ancestry proportion in any year *t* as a function of the immigration rate and an initial ancestry proportion *A*_0_. Genetic swamping is inevitable with one-way dispersal; the only stable equilibrium is *A*_*t*_ = 1 [4,10]. The expected time scale of swamping (in the absence of selection) depends entirely on the immigration rate *m*, which is the ratio of the number of immigrants to total recipient population size.

To estimate the range of downstream dispersal rates (*m*) consistent with the observed data, we conducted stochastic simulations of population growth and genetic drift according to the age-structured Brook Trout life history model [38]. To reflect the known introduction history, we first simulated one year of reproduction starting with 135 Bunches Creek fish. In the second year (corresponding to 2018) we added 135 Deep Creek and 105 Sahlee genotypes. For each subsequent year of the simulation (up to 2022, the sampling year), we added pure Bunches Creek immigrants (implicitly from the left fork population) of varying age classes using a random draw from a multinomial distribution with size *N*_*t*_*m* (where *N*_*t*_ is the total population size in the mainstem in year *t*) and probabilities determined from the stable age distribution determined from preliminary model runs (0.66 age 0, 0.21 for age 1, 0.08 for age 2, and 0.05 for age 3). We performed 10,000 replicates each for immigration rates between 0.1 and 0.25 in increments of 0.01. We then compared the distribution of simulated ancestry proportions for each immigration rate to the estimated value from our 2022 data. We defined an immigration rate as credible if the interquartile range of the simulated outcomes included the observed value of Bunches Creek ancestry in the main stem. R scripts for recreating these simulations are given in S2 File Ancestry Analysis

Because potential remediation could be undertaken in 2025 or later, we used the credible range of immigration rates to forecast the expected proportion of Bunches Creek ancestry in the mainstem population using equation 2. Thus, we use our estimated range of credible immigration rates to forecast expected future ancestry proportions in the mainstem with *A*_*0*_ set equal to our estimated value from the 2022 samples.

### Genetic diversity

To evaluate the effect of downstream dispersal on genetic diversity (SNP heterozygosity) regardless of ancestry representation, we estimated the expected heterozygosity of a mixture of source populations by pooling samples from Bunches, Deep, and Sahlee creeks in same proportion as the original translocation numbers (approximately 36%, 36%, and 27% respectively). To confirm that this mixture has greater diversity than a single source population, we performed a permutation test with the function test.g in hierfstat [39] with 10,000 permutations of the null hypothesis that diversity within populations is equivalent to the total diversity. We then compared the locus-specific diversity estimates for the pooled source populations (i.e., the original mixture in the mainstem) to the realized diversity in our samples from the mainstem and left fork of Anthony Creek (the restoration sites) using paired Wilcoxon signed rank tests with continuity correction in the stats package of R [32].

### Remediation strategies

Finally, we used these results to explore the feasibility of two strategies to recover desired genetic diversity. First, individuals from the genetically mixed mainstem population could be transported above the barrier cascade to modify the ancestry proportions of the left fork population. With continued subsequent one-way dispersal, the equilibrium ancestry proportion of the mainstem would converge on the new left fork ancestry proportion. For example, if *N*_*I*_ immigrants with average ancestry *A*_*I*_ could be transported and added to the population of *N*_*R*_ residents in the left fork, the new ancestry proportion in the left fork would be eq 3:


$$\frac{A_{I}N_{I}+N_{R}}{N_{I}+N_{R}}$$ (eq 3)

Alternatively, if the desired ancestry proportion is lower than what is possible given eq 3, then new fish could be translocated from the other original source populations (with zero Bunches Creek ancestry). In this scenario, because the ancestry proportion of the immigrants is *A*_*I*_* *= 0, the new (and expected equilibrium) ancestry proportion would be eq 4:


$$\frac{N_{R}}{N_{I}+N_{R}}$$ (eq 4)

In either case, to determine the feasibility of translocations, we required an estimate of the total population size in the left fork (*N*_*R*_).

### Population size

Along with the GRSM fisheries science team, we used population density estimates from annual three-pass depletion surveys to extrapolate a total population size for the left fork section. In 2019, 2020, 2023, and 2024 we conducted three-pass surveys of 100 m stream sections according to the electrofishing protocol described by Habera et al. [40], Kulp & Moore [19], and Kanno et al. [21]. We used 6–10 mm bar mesh nets to block the lower and upper end of each 100 m site and captured every fish encountered on three sequential passes with backpack electrofishing units. We counted and identified each fish by species, recorded mass (g) and total length (mm), and retained them in holding cages outside of the site until the depletion survey was complete. We then released all fish back into the site. We estimated abundance of each size class using the Burnham maximum likelihood estimator in the Microfish 3.0 software (Deveneter et al., 1989).

During each survey, we measured stream width at 10 m intervals to estimate the surface area of each 100 m section at the time of the survey. We used these estimates to convert abundance estimates to densities (number of fish per m^2^). We then used the total stream distance of the left fork population (711m, bounded by cascades on both ends) and the average stream width to extrapolate a total stream area (average across years 2927 m^2^). We estimated total population size by multiplying this area by the average density. While more refined population size estimates may be possible [41], this simple extrapolation is sufficient for present purposes of demonstrating an approach for planning future translocations.

## Results

### Ancestry analysis and dispersal patterns

**Fig 3:**
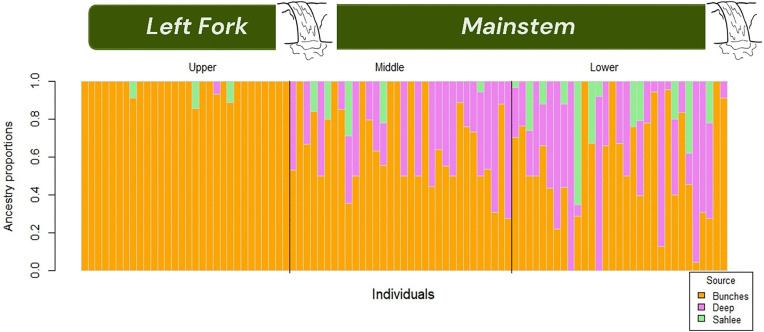
Ancestry proportions of individual Brook Trout in the restored population, estimated using HIest [36] and the estimated allele frequencies from the three source populations. Individual Brook Trout (*Salvelinus fontinalis*) are represented by vertical bars arranged along the horizontal axis according to which stream section they were captured in. The y-axis shows the proportion of genetic ancestry from each source population. Colors indicate the contribution of each source.

Ancestry estimates in the left fork stream section, where only fish from Bunches Creek were released, were 100% Bunches Creek except for four individuals with a minority of their ancestry estimated as coming from either Sahlee or Deep Creek (Fig 3). To evaluate whether those individuals are more likely to be backcrosses or mis-characterized because of estimation error, we used the classification functions in HIest (HIclass and HItest) to calculate the likelihoods of them being backcrosses, pure Bunches ancestry, or later generation backcrosses [36]. Only one individual was more likely to be a backcross than a pure Bunches Creek fish, and the difference in likelihood was small (0.09 log-likelihood units). In other words, the SNP data were not sufficiently diagnostic to reliably distinguish pure Bunches ancestry from some possible backcrosses. These results are consistent with zero upstream dispersal, but do not rule out a very low rate.

In the mainstem of Anthony Creek, Bunches Creek accounted for 63% of the total ancestry, while Deep Creek contributed 25%, and Sahlee Creek comprised 12% of the ancestry. Within the mainstem, average Bunches Creek ancestry was somewhat higher in fish collected closer to the upstream barrier (Fig. 3). While individual ancestry estimates are subject to error, we assume the population averages accurately reflect the biological processes of immigration and genetic drift.

### Genetic diversity

The gene diversity (expected heterozygosity) in the mainstem (*H*_*S*_* *= 0.07), and the left fork (*H*_*S*_* *= 0.05), were both lower than the expected diversity of the pooled source populations (*H*_*T*_* *= 0.08; S1 Table). The pooled diversity was significantly greater than within source population diversity (permutation test: *p* < 0.0001). Locus-by-locus comparisons showed significantly lower gene diversity in the mainstem relative to the pooled sources (Wilcoxon test: *V* = 2118, *p* = 0.0027), left fork relative to the pooled sources (*V* = 895, *p* < 0.0001), and left fork relative to the mainstem (*V* = 423, *p* < 0.0001).

### Migration rate estimation

**Fig 4:**
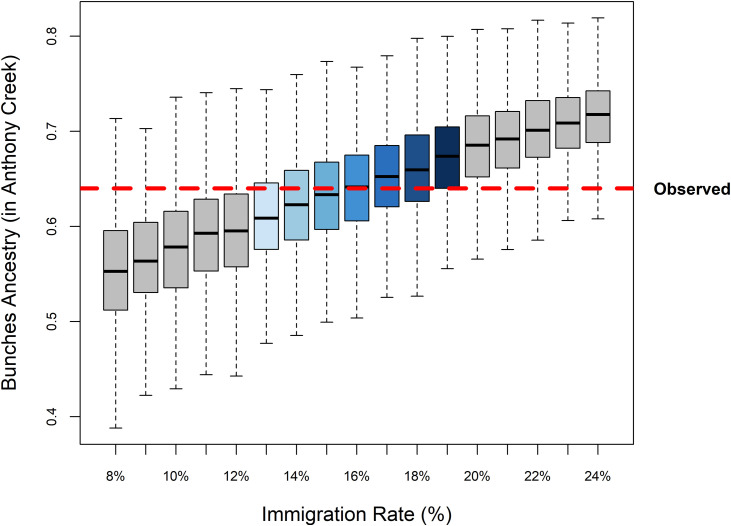
Box plot with range of immigration rates (%) on x axis and the ancestry proportion owing to Bunches Creek measured on the Y axis. The observed proportion in our sample is noted by the red dashed line. Boxes indicate the interquartile range and horizontal bars the median ancestry from 1000 simulations of each immigration rate. Whiskers extend to the furthest point within 1.5 times the interquartile range from the box.

To estimate a credible range of immigration rates from the left fork, above the barrier, to the mainstem below, we ran stochastic simulations until we found a range that fit the observed ancestry proportion of Bunches creek ancestry (63%) in the mainstem. For illustration, we declared an immigration rate credible if the interquartile range of the simulated outcomes included the observed value. Under this criterion, a suitable range of immigration rates is 13–19% (Fig 4), that is, 13–19% of the fish in the mainstem at any given time have come from the left fork in the previous year.

### Forecasting bunches creek ancestry in anthony creek

**Fig 5:**
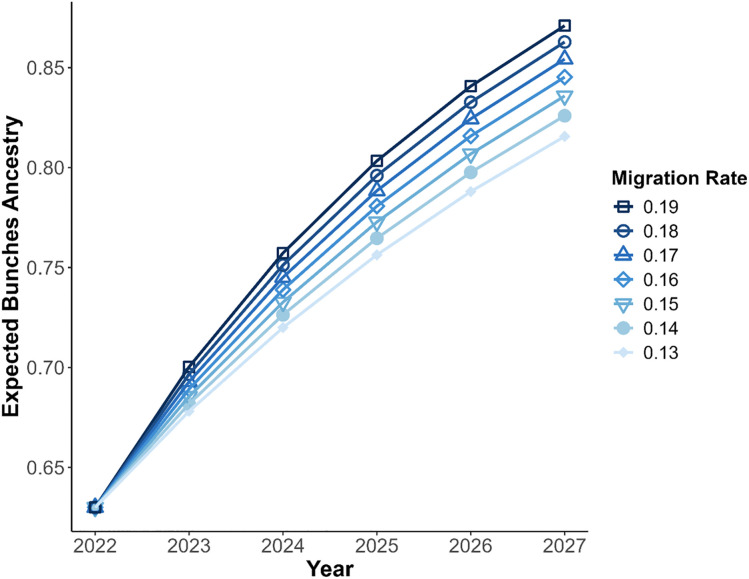
Forecasting Bunches Creek ancestry in the mainstem from the time of sample collection in 2022 to five years later in 2027.

We expect the proportion of Bunches’ ancestry in the mainstem to continue increasing without any intervention. In our five-year forecast, the mainstem of Anthony Creek is expected to reach 74–80% Bunches ancestry by 2025 and 78–87% by 2027, depending on the immigration rate (Fig 5). Using a range of migration rate estimates, we project that Bunches Creek ancestry will comprise 78–87% of the total ancestry by 2027.

### Population size

Density estimates from three-pass depletion surveys in the Left Fork increased from 1.6 fish per 100m^2^ in 2019 and 2020 to 5.1 per 100m^2^ in 2023 and 12.5 per 100m^2^ in 2024 (see S3 File Forecast Simulations). The latter is comparable to the average density of 11.6 per 100m^2^ in restored Brook Trout populations reported by Kanno et al. [21]. Extrapolating to the entire area of the Left fork (2927 m^2^), the total population size estimate for 2024 is 365 Brook Trout, and the average across years is 153.

### Remediation Strategies

Given the observed and projected ancestry proportions in the mainstem, translocating fish from the mainstem to the left fork could stabilize the system at a high fraction of Bunches ancestry. For example, if we take the mainstem ancestry in 2025 as 80% and translocate enough fish to result in a 1:1 dilution, the resulting ancestry proportion would be 90%. If feasible, this would result in doubling the population density above the barrier, but relatively minor genetic change.

To achieve roughly 1/3 contribution from each source would require translocation of new fish from Deep Creek and Sahlee Creek. The most aggressive strategy of adding enough fish from each of Deep Creek and Sahlee Creek to instantly achieve roughly 1/3 contribution from each source, would triple the population density in the Left Fork, and would probably require removing too many fish from the source populations. We used the one-way immigration model (equations 1 and 2) to illustrate a more measured approach of translocating additional fish from Deep and Sahlee Creeks multiple years in a row (Table 2), to decrease the percentage of Bunches ancestry in the system. Table 2 lists the expected effects of adding specific numbers to an assumed constant population of 365 fish. Calculations are shown in S3 File Forecast Simulations. The expected resident ancestry is expected to decay geometrically, *A*_*t*_ *=* (1 – *m*)^*t*^. The listed number per year is the total number of immigrants to be translocated into a resident population of 365 individuals. For example, if two sources were used, 45 from each source would sum to 90 immigrants and *m* = 90 ÷ (90 + 365) = 0.1978, resulting in 33.33% ancestry from each at the end of five years.

**Table 2:** Expected results of sequential translocation of a fixed fraction of individuals into a population to achieve a desired ancestry proportion of 1/3 (Immigration Fraction, m = number of immigrants ÷ (number immigrants + number of residents)).

Number Per Year	Immigration Fraction	Expected Resident Ancestry				
		Year 1	Year 2	Year 3	Year 4	Year 5
267.2	0.423	0.577	0.333			
161.4	0.307	0.693	0.481	0.333		
115.4	0.240	0.760	0.577	0.439	0.333	
89.7	0.197	0.803	0.644	0.517	0.415	0.3333

## Discussion

Our results indicate that unidirectional dispersal has disrupted the intended equal genetic contributions from the three stocked source populations in the Anthony Creek restoration site. This one-way gene flow has resulted in the mainstem’s genetic ancestry and diversity being dominated by the Bunches Creek lineage. If one-way migration continues, the restored population will trend toward genetic homogenization in terms of ancestry. Equal genetic contribution is a common goal in mixed-source reintroductions, and moving fish solely from the mainstem into the left fork is unlikely to achieve this balance. To promote a more balanced representation of the three source populations stocked in Anthony Creek, additional translocations from Deep Creek and Sahlee Creek would be required. We have not tested whether any single source harbors selectively advantageous alleles or greater adaptive potential; without that information, an equal representation of all three sources is the best recommendation to ensure a diverse gene pool, increase the restored population’s capacity to adapt to environmental changes, and enhance long-term resilience.

Distinguishing between strictly unidirectional migration and minimal movement in the opposite direction remains challenging, but our ancestry analyses confirm that upstream dispersal is negligible if not zero. The barrier between the mainstem and left fork is approximately three meters high. High seasonal water flows could theoretically facilitate upstream movement. However, our results indicate this has rarely happened in the four years between translocation and sampling. Upstream dispersal is extremely rare and thus has no chance of equalizing the genetic contributions of downstream and upstream populations. Therefore, even if limited upstream dispersal occurs, it would not meaningfully affect the conclusions or implications of this study.

Mainstem ancestry has shifted due to high immigration rates, increasing from the original 42% Bunches ancestry in 2018 to 63% at the time of sampling (2022) and a projected 74–80% in 2025. Our estimated dispersal rate of 13–19% indicates that fish are moving at a rate that significantly influences downstream populations. The precise interpretation of this estimate as a proportion of fish coming over the barrier depends on our model assumptions. For example, if fish with Bunches Creek ancestry have a fitness advantage, then a lower actual movement rate would lead to the observed outcome (vice versa if Bunches Creek fish have a fitness disadvantage). In addition, accelerated genetic drift owing to factors like over-dispersed reproductive success would expand the range of immigration rates that could be consistent with the observed outcome. Even so, the most direct interpretation of our results is that downstream dispersal has resulted in significant genetic swamping. This change highlights the importance of considering barriers and the directionality of gene flow in restoration efforts, especially when aiming for equal genetic contributions. These considerations are critical for enhancing genetic diversity in restored populations and planning for long-term resilience.

One-way migration will eventually swamp a recipient population’s ancestry [4,10]. That is not necessarily a problem if the immigrant genotypes have high fitness and the effective population size remains high. On the other hand, a high immigration rate from a lower diversity source population will tend to reduce the diversity of the recipient population, especially if the effective population size is small. Moreover, in this particular case, one-way dispersal is eliminating the intended contribution of Deep Creek and Sahlee Creek lineages and has resulted in a reduction of the overall genetic diversity in the restored population relative to the genetic diversity of a more even mixture of source lineages. Therefore, it is worth considering strategies to equalize the representation of all three lineages.

Achieving equal genetic contribution through translocating fish from the mainstem to above the barrier into the left fork is unlikely to be the best solution, albeit least taxing on management and fish. It is not an ideal solution because Bunches Creek ancestry already dominates the mainstem’s genetic makeup. The only way to attain equal genetic contribution is to translocate additional fish from Deep and Sahlee Creeks into the left fork. Previous Brook Trout studies in GRSM [21] indicated average steady state density of approximately 12 fish per 100 m^2^, which is comparable to our estimates in 2024 (S1 Dataset). This means the estimated 365 total fish in the left fork of Anthony Creek may be close to the local carrying capacity. Any additional translocations will increase population size and may push the site over what Anthony Creek can support. Therefore, it may be best to stagger translocation events over time to minimize demographic stress (Table 2).

In practice, the situation is now slightly more complex. After our sampling, additional fish from a fourth source, Bearwallow Branch, were introduced above the left fork barrier to help increase fish abundance in Anthony Creek. As a result, the upper left fork population is now approximately 50% Bunches and 50% Bearwallow Branch. If maintaining Deep and Sahlee genetic contributions across the system is a goal, additional translocations to the uppermost reaches would be necessary. If the goal is to equalize the contribution of all four source lineages, then, for example a total translocation fraction of *m* = 0.206 (split evenly between Deep and Sahlee sources) would achieve equal representation in 3 years. This approach would require careful consideration of the current population size, carrying capacity, and the number of fish that could be added without exceeding these ecological constraints.

Strategic translocation planning is critical for maximizing genetic diversity and population stability. Considerations such as carrying capacity and whether translocations should occur in a single event or be staggered over multiple years can influence restoration success. Previous research on multi-stocked restorations in GRSM has shown that, in the absence of barriers, complete, even admixture can occur between sources [38]. Here we show that dispersal barriers within a complex restoration site can have large consequences over a relatively short timescale. One-way or asymmetrical gene flow between genetically differentiated populations will transform the recipient population until it is indistinguishable from the source population. Therefore, genetic composition and management of source populations – upstream populations in the case of riverine fishes – will have outsized influence on the system as a whole.

The genetic consequences of unidirectional dispersal are particularly concerning in the context of local adaptation. Natural selection can counteract genetic swamping if the selection coefficient is greater than the immigration rate [42,43]. However, given the high immigration rates inferred in our study, only exceptionally strong selection would be capable of retaining potentially advantageous alleles from Deep Creek and Sahlee Creek. Moderately advantageous alleles, if present in the mainstem population, might be preserved by translocating fish from the mainstem to the left fork (above the barrier), where selection would be expected to increase their frequencies even if the overall contribution of Deep Creek and Sahlee Creek ancestry remains low. These dynamics underscore the importance of considering both gene flow and selection pressures when designing conservation strategies.

These findings have broad implications for conservation efforts aimed at addressing habitat fragmentation, reintroductions, and assisted migration. Asymmetric dispersal behavior can significantly shape genetic composition in populations. This effect can lead to a shift in allele frequencies along the dispersal path and, over time, cause local populations to diverge from their original genetic composition. For example, Kobayashi et al. (2024) found that one-way migration from introduced resident Rainbow Trout (*Oncorhynchus mykiss*) has reshaped native anadromous Steelhead populations. This unidirectional gene flow is shifting the receiving population toward a more resident life history, which is critical given that anadromous Steelhead are generally regarded as a higher conservation priority than resident Rainbow Trout, even within the same watershed. Such documented cases of directional change driven by asymmetric dispersal highlight a fundamental challenge in population management.

The Steelhead example [44] and our study’s findings illustrate a broader principle: applying genetic and evolutionary theory in practice is essential for refining restoration strategies and minimizing unintended genetic consequences in native fish and wildlife conservation. Our work highlights that understanding gene flow dynamics is essential for planning restoration strategies to minimize unintended genetic consequences. Careful consideration of evolutionary and population genetic principles can enhance the likelihood of creating resilient, self-sustaining populations by ensuring management interventions achieve the desired genetic outcomes.

## Conclusion

This study investigates a Brook Trout reintroduction in Anthony Creek, which serves as a natural, one-way migration system. Due to an initial stocking error, fish from a single source population were placed upstream of a barrier, while three source populations were mixed downstream. Subsequent downstream dispersal from the upstream population led to genetic swamping, such that one ancestral source dominated the downstream population.

Our analysis confirms negligible upstream dispersal and estimates a high downstream migration rate (13–19%), which has increased the upstream population’s genetic contribution in the mainstem from an initial 42% to 63% in four years. This one-way gene flow has undermined the reintroduction’s goal of creating a restored population with equal genetic contributions from all three sources.

Based on our findings, we provide management with guidance for future action. We show that simply translocating fish from the mainstem back upstream would not effectively restore the intended genetic balance. Instead, to achieve the desired genetic diversity, additional fish from the other two source populations would need to be introduced above the main barrier. These findings highlight the critical need to consider dispersal behavior, stream connectivity, and the potential for genetic swamping when designing reintroduction strategies to ensure long-term population resilience.

## Supporting information

S1 Dataset Source population genotypes, population maps, abundance estimates and analysis code. (ZIP)

S2 File Ancestry Analysis. Files and scripts for reproducing ancestry and HIEST analysis. To use the HIest function, you will first need to install the HIest_2.1.tar.gz package and then load it into your R library.
(ZIP)

S3 File Forecast Simulations. Files and scripts for reproducing forecast simulations of future populations.
(ZIP)

S1 Table Observed and Expected Heterozygosity for populations in this study. (XLSX)
